# Treatment of Acute Rheumatism

**Published:** 1887-01

**Authors:** 


### *TREATMENT OF ACUTE RHEUMATISM.*

Prof. Da Costa states that there are laid down two principal plans of treatment of acute rheumatism:

1. Salicylic acid and the salicylates. These are unquestionably the most speedy remedies, but should not be employed in those cases in which much weakness exists, for it greatly increases the sweats and depression, or in those cases where tendency to cardiac complication is manifested. In these latter it has been stated to be worse than useless.

If the acid be used, which is preferable to its salts, give not less than sixty to ninety grains in twenty-four hours. Ten grains may be given in emulsion every hour, for six hours, if borne well, and then the same doses may be given at intervals of two hours.

If the salicylates are used, give three drachms in twenty-four hours. If this plan acts at all, it will do so promptly; and if good results are not achieved by the second or third day, it had better be abandoned.

2. The alkaline plan. This consists in rapid saturation with the alkalies. It lessens the complications, but no good can be achieved by small doses. An ounce to an ounce and a half of either the bicarbonate or acetate of potassium must be given the first twenty-four hours, half as much the following day, and three or four drachms each day thereafter.

Employ until the urine becomes neutral or alkaline, and then diminish the dose as above stated.—*College and Clinical Record.*

